# Real world effectiveness and tolerability of candesartan in the treatment of migraine: a retrospective cohort study

**DOI:** 10.1038/s41598-021-83508-2

**Published:** 2021-02-15

**Authors:** Carmen Sánchez-Rodríguez, Álvaro Sierra, Álvaro Planchuelo-Gómez, Enrique Martínez-Pías, Ángel L. Guerrero, David García-Azorín

**Affiliations:** 1 Department of Medicine, University of Valladolid, Valladolid, Spain; 2 Headache Unit, Department of Neurology, Hospital Clínico Universitario de Valladolid, Avenida Ramón y Cajal 3, 47003 Valladolid, Spain; 3 Imaging Processing Laboratory, Universidad de Valladolid, Valladolid, Spain; 4 Institute for Biomedical Research (IBSAL) of Salamanca, Salamanca, Spain

**Keywords:** Neuroscience, Neurology

## Abstract

To date, two randomized, controlled studies support the use of candesartan for migraine prophylaxis but with limited external validity. We aim to evaluate the effectiveness and tolerability of candesartan in clinical practice and to explore predictors of patient response. Retrospective cohort study including all patients with migraine who received candesartan between April 2008-February 2019. The primary endpoint was the number of monthly headache days during weeks 8–12 of treatment compared to baseline. Additionally, we evaluated the frequency during weeks 20–24. We analysed the percentage of patients with 50% and 75% response rates and the retention rates after three and 6 months of treatment. 120/4121 patients were eligible, aged 45.9 [11.5]; 100 (83.3%) female. Eighty-four patients (70%) had chronic migraine and 53 (42.7%) had medication-overuse headache. The median number of prior prophylactics was 3 (Inter-quartile range 2–5). At baseline, patients had 20.5 ± 8.5 headache days per month, decreasing 4.3 ± 8.4 days by 3 months (weeks 12–16) and by 4.7 ± 8.7 days by 6 months (paired Student’s t-test, *p* < 0.001). The percentage of patients with a 50% response was 32.5% at 3 months and 31.7% at 6 months, while the retention rate was 85.0% and 58.3%. The number of prior treatments (Odds ratio 0.79, 95% CI 0.64–0.97) and the presence of daily headache (Odds ratio 0.39, 95% CI 0.16–0.97) were associated with a lower probability of response. Candesartan showed beneficial effects in the preventive treatment of migraine in clinical practice, including patients with chronic migraine, medication-overuse headache and resistance to prior prophylactics.

## Introduction

Candesartan is an angiotensin II receptor blocker (ARB) that has been used in the treatment of arterial hypertension since 1994^1–3^. In 2003^4^, the first randomized, double-blind, controlled trial with a cross-over design described that candesartan was better than placebo in the reduction of days with headache over a 12-week period. The study included patients with two to six migraine attacks per month and prior use of more than one migraine preventive. In 2014^5^, the second randomized, triple-blind, placebo-controlled, double cross-over study compared candesartan, propranolol and placebo, showing that both candesartan and propranolol achieved a statistically significant reduction of the number of days with moderate or severe headache over placebo after a 4-week period. The inclusion criteria allowed both episodic migraine (EM) and chronic migraine (CM) patients, however only one patient with CM was included, and the mean number of migraine days at baseline was 4.8. Patients with prior use of three or more migraine prophylactics were excluded.

The only real-world evidence on candesartan for migraines comes from a series of eight cases of patients from 2004 with comorbid arterial hypertension that described improvement for both migraine and hypertension^6^. Recently, in May 2020, the first retrospective cohort study was published^7^. Of the total number of patients, 84% met CM criteria and 91% had previously used prophylactic treatments. Some degree of benefit was described in 47% of the patients.

The positive results of the clinical trials led to the inclusion of candesartan in European^8^, American^9^, Danish^10^, Canadian^11^, Italian^12^, French^13^, Spanish^14^ and Hellenic^15^ guidelines, all with different degrees of recommendation. However, in Spain, candesartan is not commonly used. In a recent study, Spanish headache specialists did not include candesartan within the preferred migraine prophylactics^16^. This could be related to the fear of adverse effects, the limited evidence on CM patients, or the lack of information regarding treatment-resistant patients. Those questions were not fully addressed by the previous studies, as the sample sizes of the studies were modest: 60 patients were randomized and 46 completed one clinical trial^4^, 72 patients were randomized and 54 completed the second clinical trial^5^, and 81 patients were included in the retrospective cohort study^7^. The study population of the clinical trials included mostly EM patients, and there was no information about the effect in patients with medication-overuse headache (MOH) or prior use of prophylactic treatments^17^. We hypothesized that candesartan might be effective in many of those clinical situations. For these reasons, we aimed to evaluate the effectiveness and tolerability of candesartan in real-world practice and to explore possible predictors of a clinically relevant response.

## Methods

This is an analytic observational study with a retrospective cohort design. The study was conducted according to the Strengthening the Reporting of Observational Studies in Epidemiology (STROBE)^18^ guidelines. The study was done in the Headache Unit of Hospital Clínico Universitario de Valladolid, Valladolid, Spain. This is a tertiary academic hospital with a reference population of 280,000 patients. The headache outpatient clinic receives patients both directly from primary care and secondary care. Written informed consent was waived by ethics committee *CEIm Área de Salud Valladolid Este* (PI: 19–1451), due to the study being a review of medical records. The study was done according to the principles of the Declaration of Helsinki^19^.

### Eligibility criteria

We included patients with: (a) a diagnosis of CM or EM according to the International Classification of Headache Disorders (ICHD) operating at the time of the study^20–22^; (b) a candesartan prescription for migraine prophylaxis; and (c) an age greater than or equal to 18 years. Patients were excluded if they: (a) had a different concomitant headache disorder, except for MOH or infrequent tension-type headache (TTH)^20–22^; (b) were treated with candesartan because of arterial hypertension and did not fulfil the local standard of care for receiving migraine prophylaxis^14^; (c) had previously used candesartan; or (d) had no available records.

### Exposure

We screened the Headache Unit database, which includes information about every evaluated patient and includes data about diagnosis coded according to ICHD criteria^20–22^. The study period encompassed April 2008 to February 2019. We reviewed the use of candesartan in every migraine patient through both electronic and physical records and the electronic prescription, analysing the prior prescription of candesartan individually. In those cases, with prior candesartan use, we assessed the individual against the eligibility criteria, and if applicable, extracted the data. Recruitment was probabilistic, and all consecutive cases were evaluated.

### Variables

We evaluated demographic variables, including sex, age of migraine onset, age at the time of candesartan use, type of migraine, and, in the case of CM, months since the onset of CM. We assessed the prior history of arterial hypertension, defined by a systolic blood pressure higher than 140 mmHg and/or diastolic blood pressure higher than 90 mmHg in two separate evaluations. We evaluated the presence of other vascular risk factors and family history of migraine. We gathered information about the prior prophylactic treatments, including the name of the drug, duration of the treatment, effectiveness, and presence of adverse effects. We included prior prophylactic treatments with insufficient response and/or tolerability failure. We used the 50% responder rate, according to the International Headache Society Clinical Trials Standing Committee guidelines^23^, defined by the percent reduction in the number of migraine days or moderate or severe headache days per month compared with baseline.

Baseline was characterized by the number of monthly headache days, number of migraine days per month, number of acute medication days, and number of triptan days per month, analysing the frequency of MOH according to ICHD-3 criteria^22^. MOH was managed according to the local standard of care and did not include prior detoxification^14^. We analysed the initial dose of candesartan, the time until the therapeutic dose, the maximal dose used, the duration of the treatment, the presence of adverse effects, and treatment discontinuation.

### Study endpoints

The primary endpoint was the reduction in headache days per month between baseline (preceding 4 weeks prior to candesartan use) and weeks 8–12 after candesartan use. The secondary endpoints were the reduction in headache days per month by weeks 20–24 from baseline and the 50% and 75% responder rates at weeks 8–12 and weeks 20–24 (from baseline). We did a sub-analysis of the number of prior prophylactics, analysing patients with 0–2 compared with those with three or more prior prophylactics. We aimed to describe the patients’ profiles in which candesartan was used. We assessed the percentage of patients that discontinued the treatment and the frequency of adverse events, and we described the duration of treatment and the retention rate, defined as the percentage of patients who maintained the treatment at week 12 and at week 24. As exploratory analysis, we assessed which variables predicted a 50% response at weeks 8–12. All outcomes were evaluated on an intention-to-treat basis.

### Sample size

We used data from the study by Trovnik et al.^4^ in which, after 12 weeks of treatment, the reduction of headache days per month in patients treated with candesartan was 4.9 days higher than the placebo group (standard deviation 10.6). With a 95% confidence level, an accuracy or minimum detectable difference of 4.9 and a variance of 112.4, assuming an attrition rate of 15%, the sample size was calculated to be 86 patients for a statistical power of 80% and 116 patients for a statistical power of 90%.

### Statistics

We present nominal variables as frequencies and percentages and continuous data as means and standard deviations (SD) or medians and interquartile ranges (IQR) if the distribution was not normal determined by the Kolmogorov–Smirnov test and homogeneity of variance by using Levene test. We used a paired Student’s t-test for the primary endpoint analysis. For the secondary endpoints, we used the Fisher’s exact test to compare qualitative variables and the Student’s t-test or Mann–Whitney U test to compare continuous data, depending on the distribution. We used a Kaplan–Meier survival curve to depict the duration of treatment. For the response predictors evaluation, we used a univariate logistic regression analysis, seeking which variables were associated with a positive 50% response rate by weeks 8–12; variables with a *p* value below 0.2 were included in a multivariate regression analysis. We did a sensitivity analysis, and we repeated the multivariate regression analysis directly including all the variables in the model. To correct for multiple comparisons, we used the false discovery rate method according to the Benjamini–Hochberg procedure^24^. In all comparisons, tests were two-tailed, being accepted the statistical significance if the *p* value was < 0.05. We present odds ratios (OR) with 95% confidence intervals (CI). To address missing data, a conservative imputation technique was used (last observation carried forward) for variables with variation over time (e.g. headache days per month); and in the case of non-evolutionary variables, complete case analysis was used. The statistical analyses were done by using SPSS v26.0 (IBM Corp. Armonk, NY).

### Ethics approval and consent to participate

Clinical Research Ethics Committee CEIm Área de Salud Valladolid Este approved the study.

## Results

**Figure 1:**
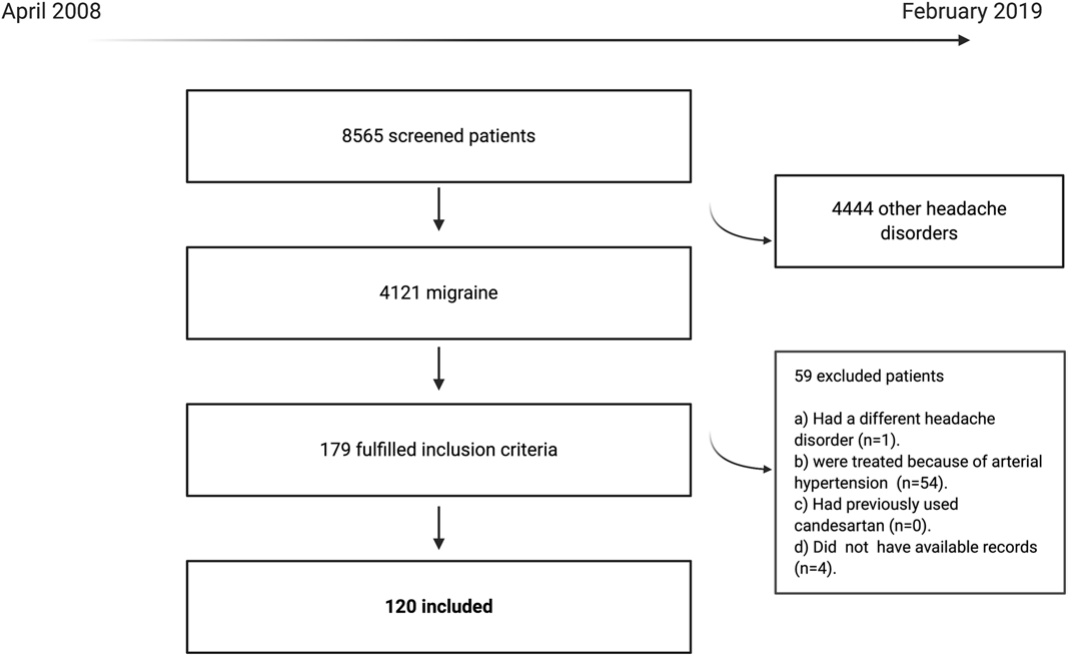
Flow-diagram of screened, included, and excluded patients.

During the study period, 8565 patients were screened, 4121 (48.1%) fulfilled the ICHD diagnosis for migraine, and 120 (2.9% of migraine patients) fit the inclusion and exclusion criteria (Fig. 1). Patients were female in 100/120 (83.3%) cases. At the time of candesartan initiation, the mean age of patients was 45.9 years (SD: 11.5). There were 36/120 (30%) patients with EM and 84/120 (70%) with CM. Frequency of vascular risk factors was 46/119 (38.7%), including arterial hypertension in 12/118 (10.2%) cases. Supplementary Table 1 summarizes the frequency of comorbidities. In 63/120 patients (52.5%) family history of migraine was referenced.

**Table 1:** Number of prior prophylactic treatments and headache days per month at baseline.

Number of prior prophylactics	n (%)
**Number of prior prophylactic drugs (n = 119)**	
0	4 (3.4%)
1	15 (12.6%)
2	17 (14.3%)
3	28 (23.5%)
4	17 (14.3%)
5	13 (10.9%)
6	11 (9.2%)
7	8 (6.7%)
8	2 (1.7%)
9	2 (1.7%)
10	0 (0%)
11	1 (0.8%)
12	0 (0%)
13	1 (0.8%)

Number of days	N (%)
**Headache days per month at baseline (n = 118)**	
0–5	1 (0.8%)
6–10	23 (19.5%)
11–15	19 (16.1%)
16–20	23 (19.5%)
21–25	8 (6.8%)
26–30	44 (37.3%)

### Baseline characterization

The mean (SD) number of preventive treatments used prior to candesartan was 3.75 (2.3). In 4/120 patients (3.3%) candesartan was the first preventive drug ever used. Table 1 shows the number of prior prophylactic treatments. The most common prior prophylactic drugs used were topiramate in 106/120 (88.3%) patients, nebivolol in 53/120 (44.1%), amitriptyline in 50/120 (41.6%), flunarizine in 49/120 (40.8%) and onabotulinumtoxinA in 46/120 (38.3%). The full list of prior prophylactics is available in supplementary materials.

In the month prior to candesartan use, the mean number of headache days per month was 20.5 (SD: 8.5), with the minimum being five and the maximum 30. There were 43/120 (35.8%) patients with daily pain. The mean number of days of symptomatic medication use was 19.0 (SD: 8.7) and the number of days of triptan use was 10.3 (SD: 7.4). MOH criteria were fulfilled by 53/120 (42.7%) patients. By the time that patients started candesartan, 35/120 (29.2%) patients were receiving another concomitant preventive treatment. Table 1 shows the distribution of patients according to the number of headache days per month they had before starting treatment.

### Candesartan use

The starting dose was 4 mg in 104/116 (89.7%) patients, 8 mg in 10/116 (8.6%) patients, and 16 mg in 2/116 (1.7%) patients. The maximum achieved dose of candesartan was 4 mg in 6/118 (5.1%) patients, 8 mg in 80/118 (66.8%) patients, 12 mg in 3/118 (2.5%) patients, and 16 mg in 29/118 (24.6%) patients. The time lapsed before doubling the starting dose was 1 week in 4/100 (4%) patients, 2 weeks in 87/100 (87%) patients, 3 weeks in 1/100 (1%) patient, 1 month in 4/100 (4%) patients, and greater than 45 and 90 days in 1/100 (1%) patient each.

### Primary endpoint

**Figure 2:**
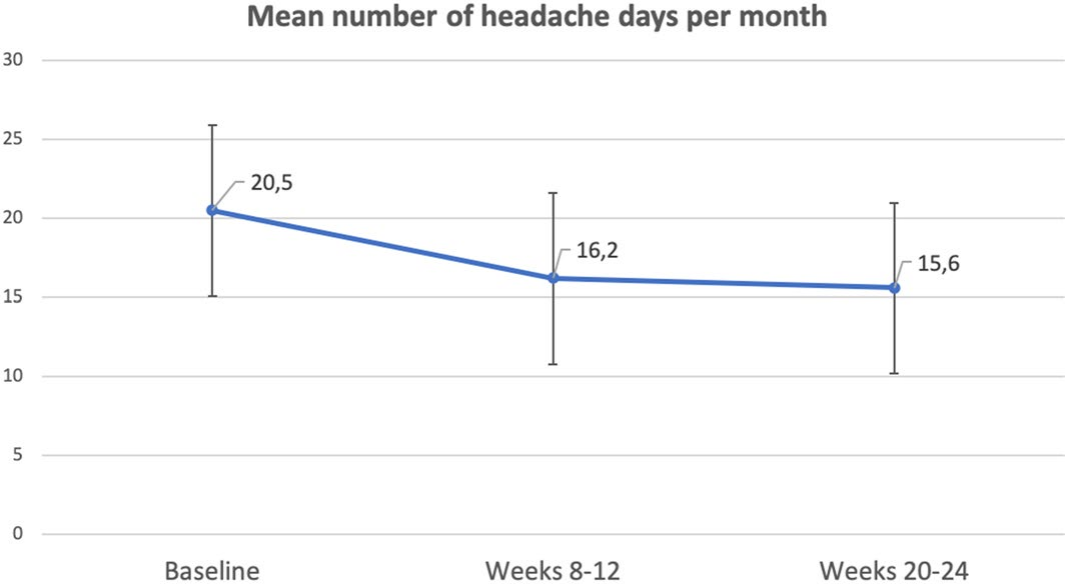
Mean (SD) number of headache days per month in the month prior to candesartan use (baseline) versus weeks 8–12 (*p* < 0.001) and versus weeks 20–24 (*p* < 0.001) after candesartan use. Paired Student’s t-test. Analysis was intention-to-treat and used the last observation carried forward imputation method.

The number of headache days per month, compared to baseline, was reduced by 4.3 days (SD: 8.4) at weeks 8–12 (*p* < 0.001), and by 4.7 days (SD: 8.7) at weeks 20–24 of treatment (*p* < 0.001). Figure 2 shows the mean number of headache days per month at baseline, after 3 months (weeks 8–12) and after 6 months (weeks 20–24) of treatment initiation.

### Secondary endpoints

**Figure 3:**
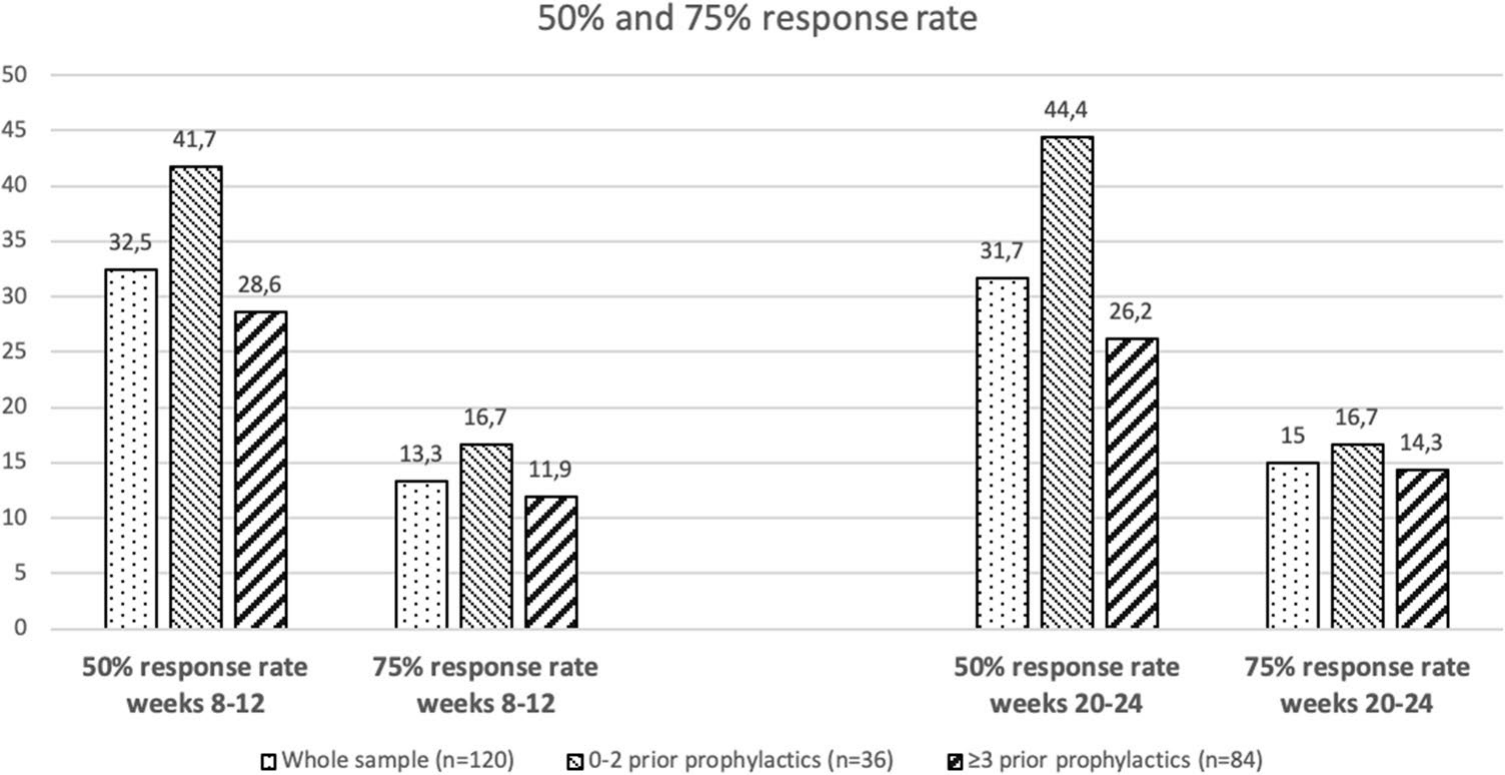
Fifty percent and 75% response rates by prophylactic category at weeks 8–12 and 20–24. Analysis was intention-to-treat and used the last observation carried forward imputation method.

The number of patients with a 50% response by weeks 8–12 was 39/120 (32.5%) and was 38/120 (31.7%) by weeks 20–24. The 50% and 75% responder rates in the whole sample and in the comparison between patients with prior use of 0–2 or three or more prophylactics are shown in Fig. 3 and supplementary table 2. No statistically significant differences were found, but a trend (*p* = 0.057) was found in the 50% responder rate at weeks 20–24.

**Figure 4:**
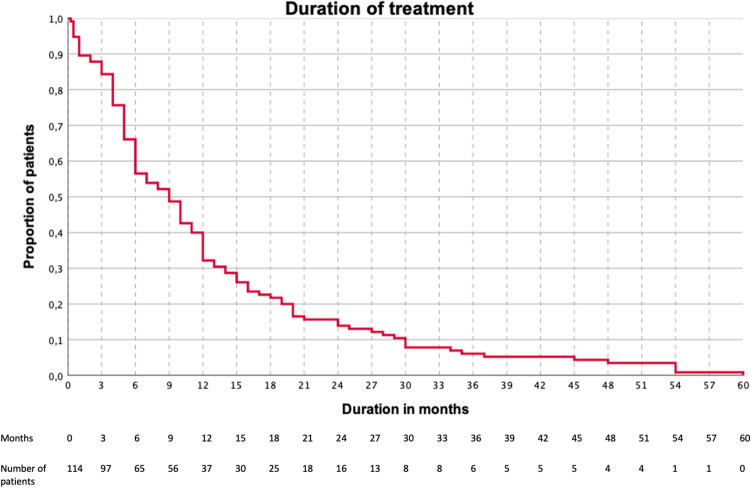
Kaplan–Meier survival curve showing the duration of candesartan treatment. X-axis, duration in months; Y-axis, the proportion of patients (n = 114).

The median duration of treatment was 9 months (IQR: 5–16), the minimum being one week and the maximum being 5 years (Fig. 4). The number of patients in which treatment was discontinued due to side effects was 22/120 (18.3%). The adverse events were light-headedness in 6/120 (5.0%) patients, hypotension in 5/120 (4.2%), sleepiness in 1/120 (0.8%), asthenia in 1/120 (0.8%), and unspecified adverse events in 6/120 (5.0%). The retention rate at week 12 was 102/120 (85.0%) and at week 24 was 70/120 (58.3%). In five (4.1%) patients, the precise duration was not available.

### Predictors of response

In the univariate regression analysis, only two variables were associated with a 50% response rate at weeks 8–12 and remained statistically significant in the multivariate regression analysis: the prior number of prophylactics (OR 0.79, 95% CI 0.64–0.97, FDR adjusted *p* value: 0.050) and the presence of daily headache (OR 0.39, 95% CI 0.16–0.97, FDR adjusted *p* value: 0.044). An odds ratio value below 1 indicates a negative association; here, patients with daily headache had 61% lower odds of responding to candesartan, and each prior prophylactic decreased the probability of response by 21%. Supplementary tables 3 and 4 show the full regression analysis of response predictors. Supplementary table 5 shows the sensitivity analysis of response predictors, with similar results.

## Discussion

The present study evaluates the effectiveness and tolerability of candesartan in a real-world setting. The main findings of the study were that candesartan showed a statistically significant reduction in the number of headache days per month, compared with baseline, and a 50% responder rate that was similar to the previous studies^4,5^. However, the treated population included treatment-resistant patients, CM sufferers, and MOH patients, with results that are in line with the only previous real-world study that addressed the effectiveness of candesartan in clinical practice. Our study contributes to the evidence provided by the previous studies, providing real-world evidence.

Concerning efficacy endpoints, the reduction in the number of headache days per month after 3 months of treatment was similar in our study (4.3 days) as in previously published pivotal studies (3.8^4^ and 2.9^5^ days). In addition, our study also assessed for the first time the efficacy at 6 months after the start of treatment, with the average reduction of 4.7 days being relevant considering that most guidelines recommend maintaining the treatment for at least 6 months 8–14). Given that the mean reduction might not be the optimal endpoint in the case of a highly variable response, we also calculated the 50% responder rate. In our study, the 50% responder rate was 32.5% after 3 months, compared to 31.6%^4^ and 43%^5^ of pivotal trials. Another remarkable result of our study is that the 75% response rate was also evaluated for the first time (13.3% at 3 months and 15% at 6 months). In the only other study done in clinical practice, the response rate considering patients who described any degree of benefit from taking candesartan was 47%^7^. However, in that study, authors judged as response the reduction in the number of headache days, migraine days or the severity of migraine attacks while using candesartan.

In our study, candesartan was mainly prescribed to patients with CM (70%), similarly to another retrospective cohort study (83.9%)^7^ with a mean number of headache days per month of 20.5, whereas in other clinical trials the sample was mainly based on EM patients (98.4%^5^ and 100%^4^), with a mean number of headache days per month of 8.2^5^ and 8.4^4^ respectively. In our sample, the mean number of prior preventive treatments was 3.7, similar to real-world studies^7^; other studies have excluded patients with failure of more than one previous preventative^4^ or more than two previous preventatives^5^.

The study includes patients from 2008 onwards. By that time, ICHD-2 was the official classification^20^, and the inclusion of patients with a different definition of CH could influence the results. However, the main difference with the ICHD-3 beta and ICHD-3 is the fact that at least 8 of the headache days per month must be migraine days^21,22^. This could influence the results by increasing the positive predictive value of the migraine diagnosis after 2013^21^. If patients with chronic tension-type headache were misclassified as migraine and treated with candesartan, the expected effect on the results would be negative, as there is no evidence on the efficacy of candesartan in other headache disorders.

We explored the 50% responder rate in patients with prior use of 0–2 migraine prophylactics, where the percentage of patients with a 50% response was 41.7% at weeks 8–12; however, we did not find statistically significant results showing that the response in those patients was higher. This could be due to a lack of statistical power given the small sample size, so larger studies are needed. Indeed, we observed the number of prior prophylactic treatments as a predictor of response. We also observed that patients with daily headache had 61% less odds of responding compared with those without daily headache, similarly to other retrospective studies where patients with daily headache had 84% lower odds^7^. The presence of daily headache has also been described as a predictor of worse response in patients treated with onabotulinumtoxinA^25,26^.

The percentage of patients who discontinued candesartan due to the presence of adverse effects was 18.3% in our sample. In clinical trials, this percentage was 5.3%^4^ and 4.9%^5^, although the usual dose was higher (16 mg). This could be related not only with the study design, but also with the geographical factor, being all our patients treated in Spain. The rate of side effects was 46.6%^4^, 50%^5^ and 30.8%^7^ in previous studies. Dizziness was the most commonly reported side effect, described in 19.3%^4^ and 36.4%^5^ of patients. Due to the retrospective nature of our study, we used the retention rate and the duration of treatment as additional surrogate markers of possible tolerability problems. In clinical trials comparing the efficacy of candesartan as an antihypertensive versus other angiotensin-converting enzyme inhibitors (ACEIs) or placebo, the adverse events rate of candesartan was 11%^27^ and 16.1%^28^.

The effect of candesartan as a preventive treatment of migraine could be related to how the renin–angiotensin–aldosterone system affects the brain. The action of those on sympathetic tone, regulation of brain flow, neurogenic inflammation, susceptibility to oxidative stress, nociception, endothelial disfunction and neurotransmitter levels have been proposed as possible mechanisms^29–31^, however, the mechanism of action as a prophylactic is not fully understood.

Two reviews conducted in 2010^32^ and 2019^33^ on the effect of ACEIs and ARBs on migraine treatment showed that these drugs may be useful as preventive treatments, especially in patients with hypertension or at high risk of side effects. In addition, patients treated with ARBs for reasons other than migraine showed a 31% lower incidence of headache during the treatment with these drugs^33^. The uniqueness of candesartan compared with other ARBs might be related to the selectivity, half-life, and tolerability^3^; however, studies on other ARBs are even more scarce^34,35^.

This study has important limitations. First, it is a retrospective study and thus in some patients there was loss of follow-up or incomplete data. We compensated by managing missing data with conservative imputation techniques and by reviewing primary care records. Our headache unit receives patients directly from primary care; however, for the present study, most patients had previously received prophylactic treatment. This was a single centre-study and the hospital was part of the public healthcare system and patients were treated at no cost, which could also affect the external validity when comparing with other countries or geographical regions. Our sample size was modest, and some secondary endpoints might be falsely negative because of lack of power. Despite of that, the inclusion period was prolonged and three different editions of the ICHD were used, which could also influence the results. Due to the observational design, there was no formal control group. In line with other real-world studies or even clinical trials assessing the effectiveness of headache therapies, we used as control the month prior to the use of candesartan. The lack of control group difficult to draw conclusions on sustained efficacy, regression to the mean effect or placebo effect of the drug. Future studies should evaluate efficacy and tolerability of candesartan in migraine prophylaxis prospectively, or ideally in randomized controlled trials including treatment-resistant patients and comparing it versus placebo or active comparators.

## Conclusion

Candesartan is a potentially effective drug in the preventive treatment of migraine. In this study, the number of headache days per month after three and 6 months of treatment was significantly lower than at baseline. A third of the treated patients experienced a decrease in the monthly number of headache days to less than half of the baseline days. Candesartan showed beneficial effects in patients with CM, with MOH, and with prior failure of other prophylactic treatments. A higher number of prior preventive treatments and patients with daily headache were associated with a lower probability of response to candesartan. A sixth of patients discontinued the treatment due to adverse events. Future studies should evaluate candesartan prospectively and ideally in randomized, controlled trials.

## Supplementary Information

Supplementary Information

Supplementary Information

Supplementary Information

Supplementary Information

Supplementary Information

## Data Availability

The datasets used and/or analysed during the current study are available from the corresponding author upon reasonable request.

## Abbreviations

ARB: Angiotensin II receptor blocker
EM: Episodic migraine
CM: Chronic migraine
MOH: Medication overuse headache
STROBE: Strenghtening the reporting of observational studies in epidemiology
ICHD: International classification of headache disorders
TTH: Tension-type headache
SD: Standard deviation
IQR: Inter-quartile range
OR: Odds ratio
CI: Confidence interval
