# Supplementary material for: Real world effectiveness and tolerability of candesartan in the treatment of migraine: a retrospective cohort study
Source: Sci Rep. 2021 Feb 15;11:3846. doi: 10.1038/s41598-021-83508-2 (PMC7884682; doi:10.1038/s41598-021-83508-2)
Supplement: Supplementary file 1 — Supplementary Information [file 41598_2021_83508_MOESM1_ESM.docx]

**Supplementary table 1**: Frequency of comorbidities by category

| Comorbidity | Present | Absent | Missing data |
| --- | --- | --- | --- |
| Vascular risk factors | 46 (38.3%) | 73 (60.8%) | 1 (0.8%) |
| Hypertension | 12 (10.0%) | 106 (88.3 %) | 2 (1.6%) |
| History of depression and/or anxiety | 52 (43.3%) | 67 (55.8%) | 1 (0.8%) |
| Insomnia | 29 (24.2%) | 88 (73.3%) | 3 (2.5%) |
| Pulmonary disorders | 12 (10.0%) | 106 (88.3 %) | 2 (1.6%) |
| Other painful conditions | 27 (22.5%) | 90 (75.0%) | 3 (2.5%) |
| Nephrolithiasis | 12 (10.0%) | 106 (88.3 %) | 2 (1.6%) |
