# Supplementary material for: Real world effectiveness and tolerability of candesartan in the treatment of migraine: a retrospective cohort study
Source: Sci Rep. 2021 Feb 15;11:3846. doi: 10.1038/s41598-021-83508-2 (PMC7884682; doi:10.1038/s41598-021-83508-2)
Supplement: Supplementary file 2 — Supplementary Information [file 41598_2021_83508_MOESM2_ESM.docx]

**Supplementary table 2:**

Fifty percent and 75% response rates by prophylactic category at two time points.

|  | All patients (n=120) | 0-2 prior prophylactics (n=36) | ≥3 prior prophylactics (n=84) | *P* value* |
| --- | --- | --- | --- | --- |
| 50% response weeks 8-12 | 39 (32.5%) | 15 (41.7%) | 24 (28.6%) | 0.203 |
| 75% response weeks 8-12 | 16 (13.3%) | 6 (16.7%) | 10 (11.9%) | 0.560 |
| 50% response weeks 20-24 | 38 (31.7%) | 16 (44.4%) | 22 (26.2%) | 0.057 |
| 75% response weeks 20-24 | 18 (15.0%) | 6 (16.7%) | 12 (14.3%) | 0.783 |

* 2-tailed Fisher’s exact test comparing the groups of patients with 0-2 prior prophylactics and ≥3 prior prophylactics. Analysis was intention-to-treat.
