# Supplementary material for: Real world effectiveness and tolerability of candesartan in the treatment of migraine: a retrospective cohort study
Source: Sci Rep. 2021 Feb 15;11:3846. doi: 10.1038/s41598-021-83508-2 (PMC7884682; doi:10.1038/s41598-021-83508-2)
Supplement: Supplementary file 3 — Supplementary Information [file 41598_2021_83508_MOESM3_ESM.docx]

**Supplementary table 3:**

Univariate logistic regression of predictors of a 50% response at weeks 8 to 12.

| Variable | Odds ratio | 95% confidence interval | Original *P* value | Benjamini-Hochberg adjusted *P* value |
| --- | --- | --- | --- | --- |
| Prior number of prophylactics | 0.773 | 0.631-0.946 | **0.013** | 0.130 |
| Presence of daily headache | 0.339 | 0.139-0.828 | **0.018** | 0.090 |
| Prior history of hypertension | 2.212 | 0.664-7.374 | 0.196 | 0.653 |
| Female sex | 0.524 | 0.197-1.395 | 0.196 | 0.490 |
| Age of candesartan use | 1.016 | 0.982-1.051 | 0.357 | 0.714 |
| Concomitant preventive treatment | 0.759 | 0.321-1.793 | 0.529 | 0.882 |
| Presence of allodynia | 0.739 | 0.264-2.064 | 0.563 | 0.804 |
| Presence of MOH | 0.683 | 0.153-3.054 | 0.618 | 0.772 |
| Age of migraine onset | 0.992 | 0.956-1.031 | 0.694 | 0.771 |
| Months of CM | 1.000 | 0.992-1.008 | 0.970 | 0.970 |

CM, chronic migraine; MOH, medication-overuse headache.
