# Supplementary material for: Real world effectiveness and tolerability of candesartan in the treatment of migraine: a retrospective cohort study
Source: Sci Rep. 2021 Feb 15;11:3846. doi: 10.1038/s41598-021-83508-2 (PMC7884682; doi:10.1038/s41598-021-83508-2)
Supplement: Supplementary file 4 — Supplementary Information [file 41598_2021_83508_MOESM4_ESM.docx]

**Supplementary table 4**:

Multivariate logistic regression analysis of predictors of a 50% response at weeks 8 to 12.

| Variable | Odds ratio | 95% confidence interval | Original *P* value | Benjamini-Hochberg adjusted *P* value |
| --- | --- | --- | --- | --- |
| Prior number of prophylactics^a^ | 0.791 | 0.644-0.970 | **0.025** | **0.050** |
| Presence of daily headache^b^ | 0.390 | 0.156-0.973 | **0.044** | **0.044** |

^a^Included as a continuous variable in this model.

^b^Included as a categorical variable in this model.
