# Supplementary material for: Real world effectiveness and tolerability of candesartan in the treatment of migraine: a retrospective cohort study
Source: Sci Rep. 2021 Feb 15;11:3846. doi: 10.1038/s41598-021-83508-2 (PMC7884682; doi:10.1038/s41598-021-83508-2)
Supplement: Supplementary file 5 — Supplementary Information [file 41598_2021_83508_MOESM5_ESM.docx]

**Supplementary table 5:**

Multivariate logistic regression of predictors of a 50% response at weeks 8 to 12 (with no prior selection of variables)

| Variable | Odds ratio | 95% confidence interval | Original *P* value | Benjamini-Hochberg adjusted *P* value |
| --- | --- | --- | --- | --- |
| Prior number of prophylactics | 0.626 | 0.429-0.913 | **0.015** | 0.075 |
| Presence of daily headache | 0.256 | 0.071-0.923 | **0.037** | 0.123 |
| Prior history of hypertension | 1.158 | 0.1-13.458 | 0.907 | 0.907 |
| Female sex | 0.541 | 0.083-3.530 | 0.521 | 0.744 |
| Age of candesartan use | 0.924 | 0.822-1.039 | 0.185 | 0.370 |
| Concomitant preventive treatment | 0.721 | 0.179-2.901 | 0.645 | 0.806 |
| Presence of allodynia | 1.143 | 0.277-4.707 | 0.853 | 0.948 |
| Presence of MOH | 0.12 | 0.028-0.509 | **0.004** | **0.04** |
| Age of migraine onset | 1.034 | 0.976-1.096 | 0.259 | 0.432 |
| Months of CM | 1.011 | 0.999-1.022 | 0.066 | 0.165 |

CM, chronic migraine; MOH, medication-overuse headache.
